# Phytochemical analysis for ten Peruvian *Mentheae* (*Lamiaceae*) by liquid chromatography associated with high resolution mass spectrometry

**DOI:** 10.1038/s41598-023-37830-6

**Published:** 2023-07-03

**Authors:** Carlos A. Serrano, Gretty K. Villena, Eric F. Rodríguez, Belea Calsino, Michael A. Ludeña, Gari V. Ccana-Ccapatinta

**Affiliations:** 1 Laboratorio de Química Orgánica, Universidad Nacional de San Antonio Abad del Cusco, Cusco, Peru; 2 Laboratorio de Micología y Biotecnología, Universidad Nacional Agraria La Molina, Lima, Peru; 3 Herbarium Truxillense (HUT), Universidad Nacional de Trujillo, Trujillo, Peru; 4 Centro de Salud MINSA de Pisaq, Cusco, Peru; 5 Farmacia Magistral Solidaridad, Cusco, Peru

**Keywords:** Drug discovery, Plant sciences

## Abstract

The profile of secondary metabolites in ten members of tribe *Mentheae* (*Nepetoideae*, *Lamiaceae*) from Peru by liquid chromatography associated with high resolution mass spectrometry, is presented. Salvianolic acids and their precursors were found, particularly rosmarinic acid, caffeic acid ester derivatives, as well as a diversity of free and glycosylated flavonoids as main substances. At all, 111 structures were tentatively identified.

## Introduction

The tropical Andes are considered one of the most diverse areas on the planet in terms of vascular plants. The flora of Perú is extremely rich, and its territory is home to some 25,000 species, almost 10% of all plants in the world. However, the percentage of them scientifically studied is quite low^1^. Phytochemical research on Peruvian biodiversity proved to be fundamental in the development of modern medicine, e.g. the isolation of cocaine from *Erythroxylum coca* was a milestone in the development of local anesthetics^2^, similarly the isolation of the first antimalarial agent, quinine from *Cinchona ledgeriana* cortex initiated “the alkaloids golden age”^3^. Most of those phytochemical investigations were conducted overseas, a fact that reflects the absence or the restricted access of resources and infrastructure for developing classical phytochemical research in Peru. Today, modern platforms maybe applied for the metabolic characterization of Peruvian flora, a task that can be achieved by a liquid chromatography associated with high resolution mass spectrometry (LC-HRMS) method since it is less time consuming compared to classic methods of isolation and structure identification. Some recent investigations that exemplify the use of LC-HRMS for describing the phytochemical profile of Peruvian flora include the metabolic profile on medicinal plants of the genus *Chuquiraga* (*Asteraceae*)^4^ and that related to *Capsicum* (*Solanaceae*) fruits^5^.

Perú has several traditional medicine systems, that of the northern Andes^6,7^, that of the southern Andes^8^ and that of the Amazonian forest^9^, each one of them with its main and minor plants and particular practices. With the passage of time, those traditional medicines are getting combined a fact that is especially noticeable in Lima city, the capital of Peru^10^. One aspect that is worth to highlight is that, especially in Andean medicines, but not in Amazonian ones, there is an important contribution of plants belonging to the *Lamiaceae* family to the traditional medicine systems.

The large family *Lamiaceae* has twelve subfamilies. The *Nepetoideae* subfamily, with 3400 species and 105 genera, has three tribes^11^: *Elsholtzieae*, *Ocimeae* and *Mentheae*, the latter with 65 genera. The *Mentheae* tribe is chemically characterized by having volatile terpenoids and a phenolic acid called rosmarinic acid that makes these plants aromatic and with medicinal properties^12,13^ *Mentheae* can also be classified into 3 subtribes: *Menthinae* (43 genera), *Salviinae* (10 genera) and *Nepetiinae* (12 genera)^14,15^. In Peru (Herbario Nacional Universidad de San Marcos-Perú, October 2017), the main genera of *Mentheae* were *Clinopodium* (29 species), *Hedeoma* (1 specie), *Lepechinia* (11 species), *Minthostachys* (7 species) and *Salvia* (60 species). *Clinopodium*, *Hedeoma*, and *Minthostachys* belong to the *Menthinae* subtribe, while *Lepechinia* and *Salvia* belong to the *Salviinae* subtribe. Investigations on the non-volatile components in Peruvian *Mentheae* are relatively scarce compared to the works related to essential oils^16^ . In a previous work^17^ the contents of rosmarinic acid, triterpenic acids, oleanolic and ursolic were quantified in thirteen Peruvian *Mentheae*. The highest content of rosmarinic acid was observed in *Lepechina meyenii* (Walp.) Epling and the highest content of triterpenic acids in *Clinopodium revolutum* (Ruiz & Pavón) Govaerts. Subsequently^18^, the non-volatile compounds were unambiguously or reasonably identified in two *Lepechinia* species: *L. meyenii* and *L. floribunda* (Benth.) Epling, by LC-HRMS, where the presence of salvianolic acids and diterpenoids were notable.

LC-HRMS methods have been used to comprehensively analyze the phenolic components of plants, this implies procedures for the systematic manually identification of mass spectra^19,20^ and also the use of suitable software^21,22^, in both cases the procedure involves recording of diagnostic ions for classification and then the identification of characteristic ionic products and neutral losses for confirmation. In the present communication, the profile of secondary metabolites by LC-HRMS is reported for ten Peruvian *Mentheae*: *Clinopodium* (4 species), *Salvia* (4 species), *Hedeoma* (1 species) and *Minthostachys* (1 species).

## Results

### Phytochemical profile

**Figure 1:**
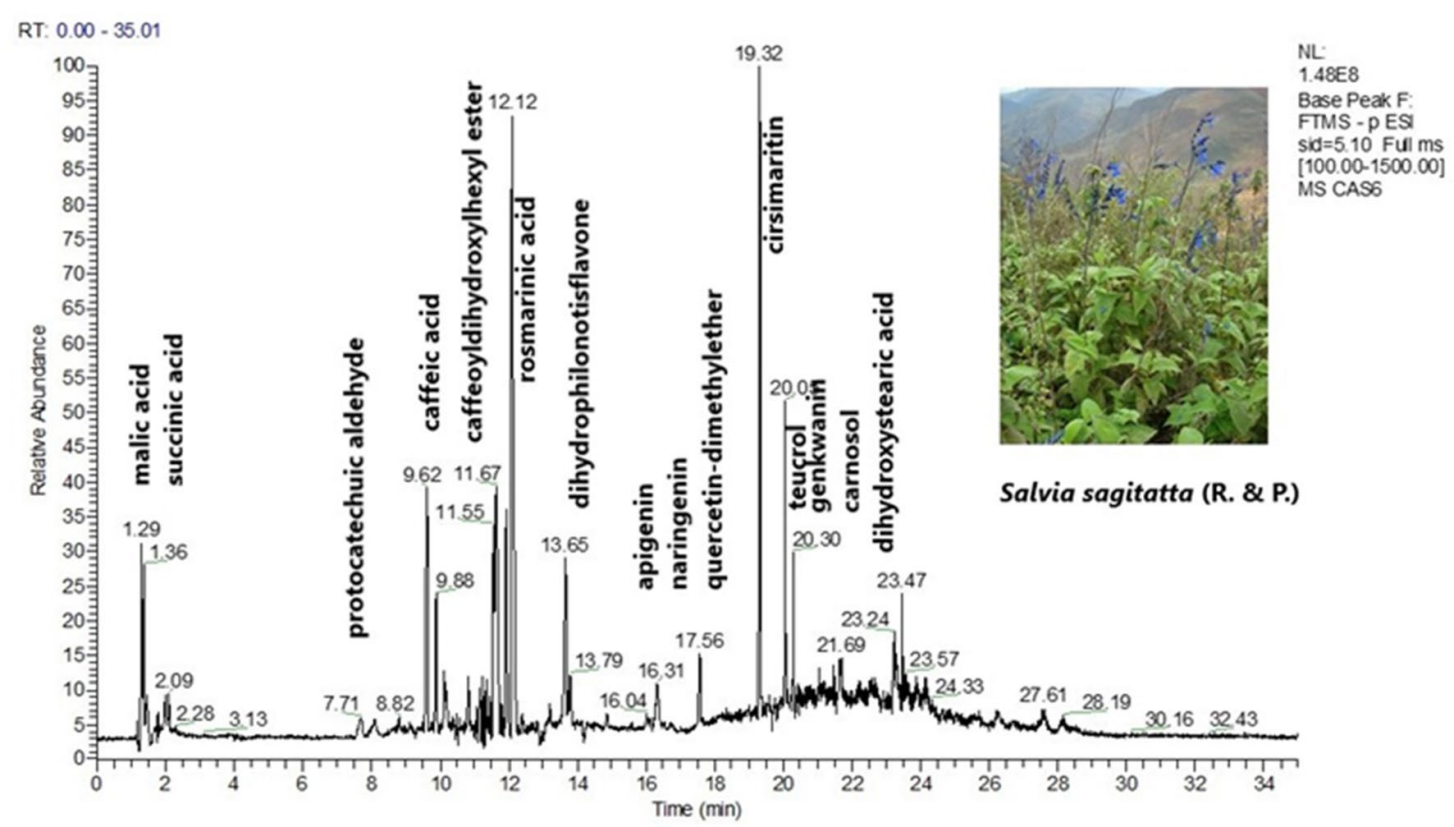
ESI (−) chromatogram of *Salvia sagitatta*.

**Figure 2:**
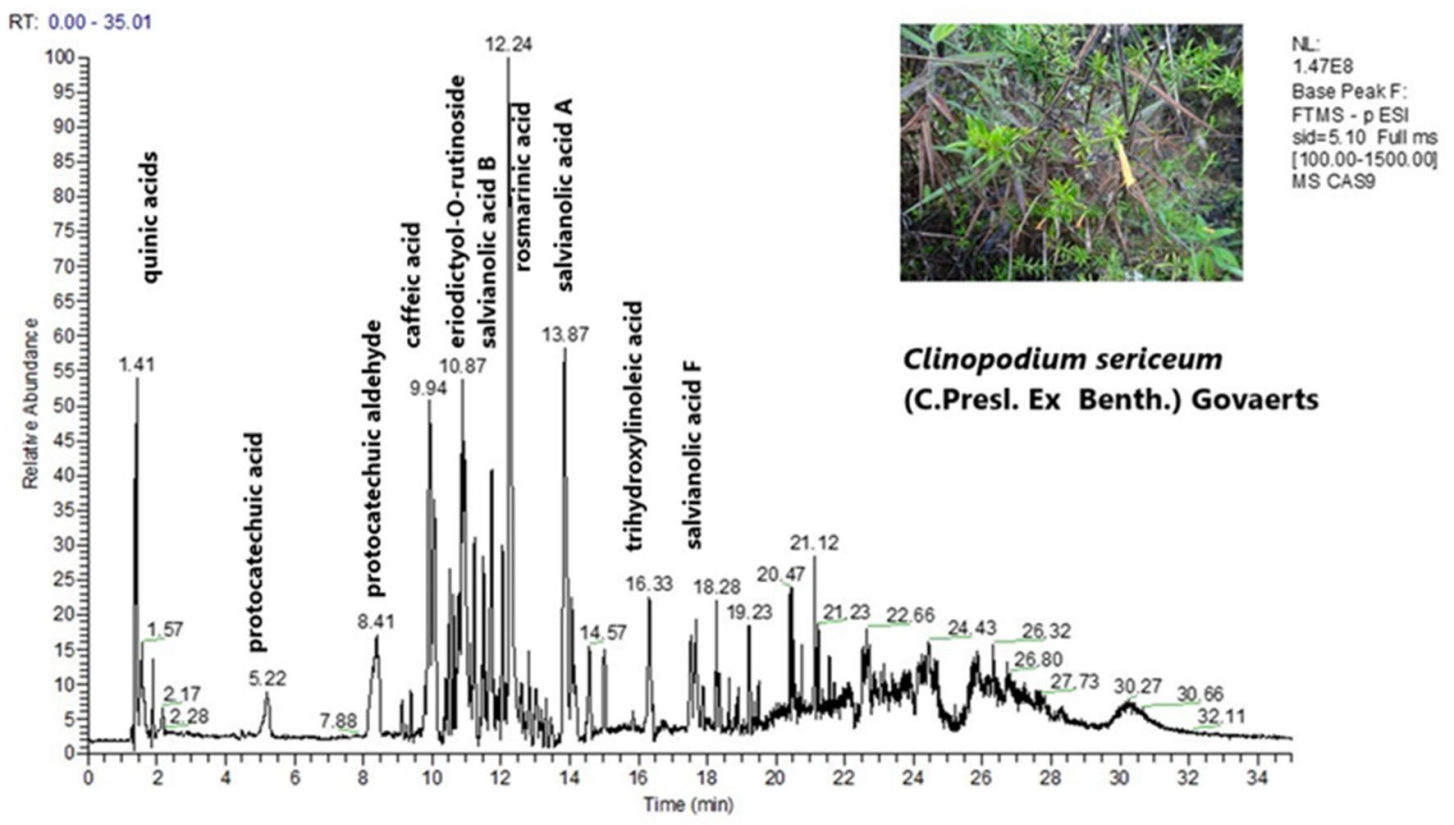
ESI (−) chromatogram of *Clinopodium sericeum*.

**Figure 3:**
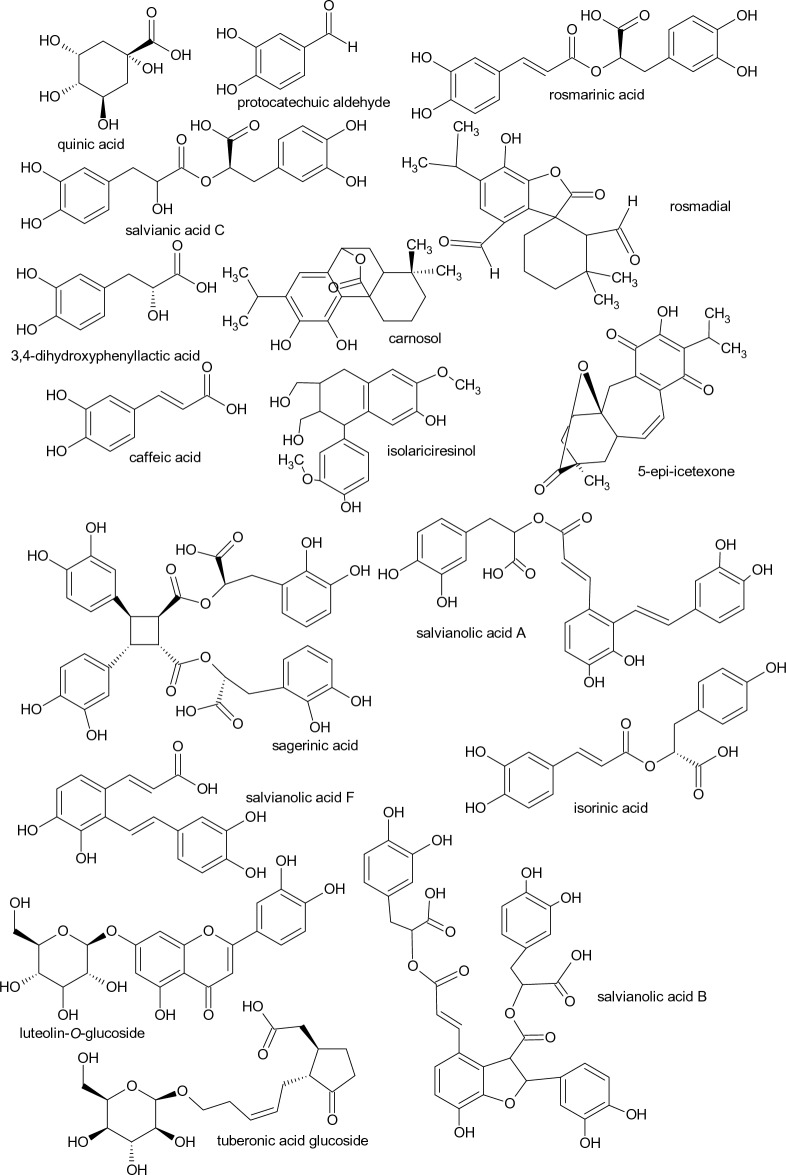
Chemical structures of main metabolites identified.

The LC-HRMS metabolite profile of the ethanolic extracts of the ten peruvian *Mentheae* was obtained in the negative mode (ESI (−)) and the detected compounds appear in Table 1. Assignments were made based on the literature^21–37^. Isomers of quinic acid (*m/z* 191.0556), danshensu (*m/z* 197.0450), protocatechuic aldehyde (*m/z* 137.0239), and caffeic acid (*m/z* 179.0350) occur in most plants. Equally abundant are the monocaffeoylquinic acids present in seven species. *Minthostachys mollis* contains four different monocaffeoylquinic acids. Several derivatives of ferulic acid and *p*-coumaric acid could also be identified. The 4 (para) substitution or the 3,4 substitution with respect to C_3_ cannot be determined by MS, however this is the substitution reported in *Mentheae*^19,20,23,38–49^. Caffeic acid, protocatechuic aldehyde and protocatechuic acid share the same substitution pattern. Furthermore, a diversity of flavonoids (flavonols, flavones, flavanones, flavanonols) was found in all the samples, both free and glycosylated. *Minthostachys mollis*, *Clinopodium sericeum* and *Clinopodium pulchellum* are the most diverse with respect to their flavonoids. The most frequent flavonoid aglycones were luteolin (*m/z* 285.0404), quercetin (*m/z* 301.0354), kaempferol (*m/z* 285.0404) and apigenin (*m/z* 269.0455). Eupatorin is present in five of the species studied^50^. In *Clinopodium revolutum,* apigenin and luteolin C- hexosides were detected. In all the samples the presence of rosmarinic acid (*m/z* 359.0772) was detected. In *Clinopodium revolutum*, salvianic acid C (*m/z* 377.0881), which is the result of hydrating the double bond of rosmarinic acid, has been detected, and, in *Salvia sagitatta*, teucrol (*m/z* 315.0880)^51^, a decarboxylated rosmarinic acid was observed. Isorinic acid (*m/z* 343.0827) a rosmarinic acid molecule without the 3-OH was present in *Clinopodium brevicalyx*, *Salvia sagitatta*, *Salvia cuspidata* and *Hedeoma mandoniana*. Methyl (*m/z* 373.0931) and ethyl (*m/z* 387.1088) esters of rosmarinic acid were present in *Salvia cuspidata* and *Clinopodium brevicalyx*. In *Salvia cuspidata* and *Clinopodium revolutum*, the dimer of rosmarinic acid, sagerinic acid (*m/z* 719.1598), which is a molecule with a stabilized cyclobutane ring, was found. *Clinopodium pulchellum* displayed the presence of salvianolic acid A (*m/z* 493.1143) and salvianolic acid F (*m/z* 313.0722). In *Clinopodium brevicalyx, Clinopodium sericeum* and *Hedeoma mandoniana*, the presence of salvianolic acid B (*m/z* 717.1443) was observed, a particularly important substance due to its effect on neurodegenerative diseases^52^. However, the plant with the greatest diversity of salvianolic acids was *Clinopodium sericeum*, "romero de jalca", in addition to salvianolic acid B, lithospermic acid (*m/z* 537.1038), two isomers of salvianolic acid A and two isomers of salvianolic acid F. This type of substances is very important due to its effect on cell fibrosis (scar formation) in direct relation to cancer^53^. Among the other substances found, it should be noted that the *Rosmarinus* type diterpenoids, common in *Lepechinia*^18,54^ are scarce in this work; only *Salvia sagitatta* and *Salvia cuspidata* show the presence of carnosol (*m/z* 329.1761) and the phenolic diterpenoid, rosmadial (*m/z* 343.1552) in the last one^28^. *Salvia haenkei* contains the ent-(*5R,9R*)-15,16-epoxy-*10S*-hydroxycleroda-3,7,13(16),14-tetraene-17,12S; 18,19 diolide (*m/z* 355.1190)^26^, while *Salvia cuspidata* had a lignan, isolariciresinol (*m/z* 359.1502) previously reported in *Linum* seeds^31,55^, and 5-epi-icetexone (*m/z* 341.1396) described as an anti- *Trypanosoma cruzii* molecule^56^. Oleanolic and ursolic triterpenic acids, quantified in a previous report by Serrano et al.^17^, do not appear in this analysis due to the elution program used, which does not reach 100% acetonitrile^57^. Figure 1 shows the typical ESI (−) chromatogram of *Salvia sagitatta* and Fig. 2 shows the chromatogram of *Clinopodium sericeum*. The chemical structures of the main metabolites detected are displayed in Fig. 3.

**Table 1:** Compounds detected in the ethanolic extracts of then Peruvian *Mentheae* by LC-HRMS.

No peak	Assignment	Rt	[M–H]^−^	Experimental mass	Δ (ppm)	Fragments	Detected in*	References
1	Quinic acid	1.33	C_7_H_11_O_6_	191.0559	1.57	127.0394	*Cb, So, Mm, Sc, Cr, Cs, Cp*	^23^
2	Malic acid	1.36	C_4_H_5_O_5_	133.0139	1.5		*Ss, Sc,*	^23^
3	Quinic acid isomer	1.44	C_7_H_11_O_6_	191.0560	2.09	127.8695	*Cb, Mm, Cr, Cs, Cp*	^23^
4	Citric acid	1.77	C_6_H_7_O_7_	191.0196	2.09	111.0081	*So*	^23^
5	Pyroglutamic acid	1.87	C_5_H_6_O_3_N	128.0348	0.00		*Sh*	^23^
6	Succinic acid	1.98	C_4_H_5_O_4_	117.0187	0.85		*So, Mm, Ss, Sc, Cr, Cs, Sh, Hm*	^23^
7	Monoacetylglycerol	2.09	C_5_H_9_O_4_	133.0502	0.75		*Ss*	
8	Mesaconic acid	2.96	C_5_H_5_O_4_	129.0190	1.55		*Cp*	
9	3,4-dihydroxyphenyl lactic acid “danshensu”	4.05	C_9_H_9_O_5_	197.0454	2.02	123.0445, 135.0446, 179.0346	*So, Cb, So, Mm, Sc, Cr, Cs, Hm, Sh*	^24,25^
10	Protocatechuic acid	4.64	C_7_H_5_O_4_	153.0190	1.31	109.0289, 135.0448	*So, Sc, Hm, Cp*	^24^
11	1-*O*-Caffeoylquinic acid	6.39	C_16_H_17_O_9_	353.0883	2.83	135.0447, 179.0347, 191.0559	*Mm*	^58,59,58–60^
12	Protocatechuic aldehyde	7.73	C_7_H_5_O_3_	137.0239	0.00	109.0289	*Cb, So, Mm, Ss, Cr, Cs, Sh, Hm, Cp*	^24^
13	Hydroxyheptandioic acid	8.78	C_7_H_11_O_5_	175.0611	2.28		*So, Ss*	
14	*p*-Coumaroyl quinic acid	8.83	C_16_H_17_O_8_	337.0934	2.96	119.0496, 163.0398, 173.0453, 191.0559	*Mm*	^58,59,58–60^
15	3-*O*-Caffeoylquinic acid	8.99	C_16_H_17_O_9_	353.0883	2.83	173.0453, 179.0559, 191.056	*Mm, Cr*	^58,59,58–60^
16	Caffeic acid *O*-hexoside	9.02	C_15_H_17_O_9_	341.0883	2.93	179.0347, 233.0458, 251.0564, 281.0670	*So, Ss, Sc*	^60,41^
17	*p*-Coumaric acid	9.28	C_9_H_7_O_3_	163.0399	2.45	119.0497	*Ss*	^38^
18	5-*O*-Caffeoylquinic acid	9.41	C_16_H_17_O_9_	353.0883	2.83	135.0446, 179.0346, 191.055	*Cb, So, Mm, Cr, Hm, Cp*	^58,59,58–60^
19	Eucomic acid	9.54	C_11_H_11_O_6_	239.0561	2.09	195.0660, 178.0586	*Cb*	
20	Caffeic acid	9.64	C_9_H_7_O_4_	179.0348	1.67	135.0446, 161.0446	*So, Mm, Ss, Sc, Cr, Cs, Sh,*	^38,39,41^
21	Caffeic acid *O*-hexoside	9.77	C_15_H_17_O_9_	341.088	2.05	179.0345, 235.0453, 251.0561, 281.0667	*Sc*	^60,41^
22	Tuberonic acid hexoside	9.86	C_18_H_27_O_9_	387.1665	2.58	101.5668, 163.0033, 206.9725	*So, Ss, Cr, Sh*	^23^
23	*p*-Coumaroylquinic acid isomer	10.1	C_16_H_17_O_8_	337.0934	2.97	163.0397, 173.0454	*So, Mm*	^58,59,61,27^
24	Salvianic acid C	10.21	C_18_H_17_O_9_	377.0882	2.39	161.0240, 179.0347, 359.0776	*Cr*	^41^
25	*p*-Coumaroyl hexoside	10.38	C_15_H_17_O_8_	325.0930	1.85	119.0496, 163.0396	*Sc*	^60^
26	Feruloylquinic acid	10.38	C_17_H_19_O_9_	367.1040	3.0	149.0240, 191.0560, 193.0504, 173.0453	*Mm*	^20,27^
27	Quercetin 3,7-di-*O*-hexoside	10.43	C_27_H_29_O_17_	625.1407	0.32	121.0288, 179.0346, 273.0980, 301.0354, 303.1084, 463.0882,	*Cs, Cp*	^62,29,30^
28	4-*O*-Caffeoylquinic acid	10.45	C_16_H_17_O_9_	353.0880	1.12	135.0445, 179.0345, 191.0557	*Sc*	^58,59,58–60^
29	*p*-Coumaroyl hexoside	10.59	C_15_H_17_O_8_	325.0930	1.85	119.0496, 163.0396	*Sc*	^60^
30	Salvianic acid C isomer	10.67	C_18_H_17_O_9_	377.0881	2.12	197.0453, 347.1708, 359.0775	*Cr*	^41^
31	Tuberonic acid	10.67	C_12_H_17_O_4_	225.1132	2.22	134.8648, 146.9382, 168.8359, 187.9417, 213.0961	*Mm, Sh,*	^23^
32	Quercetin *O*-rutinoside	10.78	C_27_H_29_O_16_	609.1458	0.33	121.0289, 179.0345, 301.0356, 273.0881	*Mm, Cp*	^60,29,30,35^
33	Eriodictyol *O*-rutinosise	10.89	C_27_H_31_O_15_	595.1661	0.34	151.0397, 287.0562	*Cs, Sh, Hm*	^42,29,30^
34	Luteolin *O*-rutinoside	11.00	C_27_H_29_O_15_	593.1504	0.51	285.0403, 447.0928	*Cb, Sc, Cr, Cp*	^16,63,26^
35	Apigenin *O*-rutinoside	11.01	C_27_H_29_O_14_	577.1556	0.35	269.1030	*Sc, Cr*	^16,38,63,26^
36	Kaempferol *O*-hexoside	11.02	C_21_H_19_O_11_	447.0936	1.78	151.0031, 285.0406,	*Cb, Ss, Cs*	^20,29,30^
37	Quercetin *O*-hexoside	11.02	C_21_H_19_O_12_	463.0886	1.94	301.0358	*So, Mm, Ss, Sc, Cp*	^60,44,29,30^
38	Quercetin *O*-glucuronide	11.09	C_21_H_17_O_13_	477.0679	2.1	301.0356	*So, Ss*	^44,29,30^
39	Feruloyl hexoside	11.12	C_16_H_19_O_9_	355.1036	1.97	149.0240, 193.0502	*Sc*	^60^
40	Pentahydroxy-methoxyflavone hexoside	11.12	C_22_H_21_O_13_	493.0989	1.42	162.8387, 163.0397, 331.0827, 315.1089	*Cs*	^29,30^
41	Isorhamnetin *O*-hexoside	11.23	C_22_H_21_O_12_	477.1046	2.72	315.0824, 357.0352, 462.0768	*Ss*	^43,29,30^
42	Naringenin *O*- rutinoside	11.33	C_27_H_31_O_14_	579.1714	0.00	151.0030, 271.0612	*Mm, Cb, Cs, Hm, Cp*	^29,30^
43	Eriodictyol *O*- rutinoside	11.4	C_27_H_31_O_15_	595.1665	0.34	151.0033, 287.0564	*Cs*	^29,30^
44	Luteolin *O*-glucuronide	11.56	C_21_H_17_O_12_	461.0729	1.95	133.0290, 151.0395, 285.0407,	*So, Ss, Cr, Sh*	^44,29,30^
45	Luteolin *O*-hexoside	11.57	C_21_H_19_O_11_	447.0937	2.01	151.0398, 241.1084, 285.0407	*Mm, Cr*	^60,29,30^
46	Dihydrobaicalin	11.57	C_21_H_19_O_11_	447.0936	1.79	271.0250, 403.1613	*Sc*	^62^
47	Sagerinic acid	11.58	C_36_H_31_O_16_	719.1600	1.67	161.0239, 179.0348, 359.0715, 539.1186	*Sc, Cr*	^38,39,45–47^
48	Hesperetin 7-*O*-rutinoside	11.64	C_28_H_33_O_15_	609.1819	0.16	151.0397, 179.0347, 301.0718, 257.1035	*Mm, Cb, Hm, Cp*	^29,30,64^
49	Apigenin *O*-rutinoside	11.66	C_27_H_29_O_14_	577.1557	0.17	225.1129, 269.0453	*Cr*	^29,30^
50	Dimethylrosmarinic acid	11.67	C_20_H_19_O_8_	387.1091	2.84	179.0347, 135.0447, 161.0452	*So, Sh*	
51	Isorhamnetin 3-*O*-glucuronide	11.7	C_22_H_19_O_13_	491.0834	1.62	151.0396, 179.0346, 302.0388, 300.0602, 301.0358, 299.0565, 315.0513	*So*	^29,30^
52	Salvianolic acid A isomer	11.75	C_26_H_21_O_10_	493.1142	1.42	179.0344, 197.0450, 269.0821, 295.1192, 313.0723, 359.0778	*Cs*	^24,25,41,49^
53	Tetrahydroxy-methoxyflavone *O*-hexoside	11.78	C_22_H_21_O_12_	477.1041	1.68	162.8398, 163.8391, 315.1451	*Cr*	^29,30^
54	Trihydroxymethoxyflavone *O*-hexoside	11.89	C_22_H_21_O_11_	461.1093	1.95	299.0559	*Sh,*	^29,30^
55	Salvianolic acid B isomer	11.99	C_36_H_29_O_16_	717.1443	1.81	321.0616, 519.0945	*Cs*	^25,40,41,47,48^
56	Apigenin C-hexoside	12.01	C_21_H_19_O_10_	431.0984	0.00	269.0452, 281.1024, 311.1130, 341.1960, 371.1002	*Cr*	^20^
57	Rosmarinic acid	12.04	C_18_H_15_O_8_	359.0775	2.23	161.0240, 179.0345, 197.0452	*Cb, So, Mm, Ss, Sc, Cr, Cs, Sh, Hm, Cp*	^25,38,39,45,46,48^
58	Luteolin C-hexoside	12.07	C_21_H_19_O_11_	447.0934	2.91	285.0404, 297.1353, 357.1921, 387.1160	*Cr*	^20^
59	Dicaffeoylquinic acid	12.17	C_25_H_23_O_12_	515.1194	0.19	135.0444, 161.0238, 179.0345, 353.0881	*Cr*	^58,59,58–60^
60	Salvianolic acid B isomer	12.53	C_36_H_29_O_16_	717.1443	1.81	295.0611, 321.0408, 339.0512, 493.1137, 519.0930, 537.1024	*Cb, Ss, Hm*	^25,40,41,47,48^
61	Luteolin *O*-acetylhexoside	12.71	C_23_H_21_O_12_	489.1039	1.23	133.0289, 151.0395, 241.0537, 257.1035, 267.0667, 285.0404, 447.0935	*Cr*	^63,26^
62	Artemetin	12.8	C_20_H_19_O_8_	387.1089	2.32	327.1241, 342.1067, 357.0992, 372.1184	*Sh,*	^29,30^
63	Isorinic acid	12.95	C_18_H_15_O_7_	343.0827	2.62	161.0241, 327.2181	*Cb, Ss, Sc Hm*	^41,65^
64	Lithospermic acid	13.03	C_27_H_21_O_12_	537.1038	0.93	295.0610, 493.1147	*Cs*	^24,41^
65	Isosakuranetin *O*-rutinoside	13.15	C_28_H_33_O_14_	593.1874	0.51	285.0770, 594.1905	*Mm, Cp*	^29,30^
66	Methyl rosmarinate	13.28	C_19_H_17_O_8_	373.0935	2.95	179.0347, 194.0540, 359.0778	*Sc*	^20,25,38^
67	Quercetin *O*-(*p*-coumaroyl)-hexoside	13.51	C_30_H_25_O_14_	609.1242	0.49	301.0719, 447.0940, 462.0747, 594.1343	*Cr*	^29,30^
68	Eriodictyol	13.6	C_15_H_11_O_6_	287.0563	2.44	107.0133, 135.0445, 151.0030	*Cb, Cp*	^29,30^
69	Luteolin	13.62	C_15_H_9_O_6_	285.0408	3.16	133.0289, 151.0032, 241.1085	*Cb, Hm*	^29,30,32^
70	Dihydrophilonotisflavone	13.63	C_30_H_19_O_12_	571.0883	1.05	133.0290, 151.0033, 285.0410, 286.0441	*So, Ss*	^29,30^
71	Ferulic acid	13.68	C_10_H_9_O_4_	193.0504	1.55	134.0367, 149.0239, 178.0220	*Sc*	^20,25,66^
72	Salvianolic acid A isomer	13.87	C_26_H_21_O_10_	493.1141	1.22	159.8595, 179.0345, 197.0451, 295.0612, 269.0821, 313.0719, 359.0774	*Cs, Cp*	^24,25,41,49^
73	Protocatechuic acid *O*-(hydroxybenzoyl)hexoside	13.94	C_20_H_19_O_11_	435.0935	1.61	137.0239, 153.0191, 297.1346, 315.1452	*Cr*	
74	Trihydroxy-methoxyflavone	14.00	C_16_H_11_O_6_	299.0565	3.01	151.0397, 255.0698, 285.0413	*Ss, Sh*	^29,30,32,35^
75	Hesperetin *O*-hexoside	14.27	C_22_H_23_O_11_	463.1250	2.06	151.0395, 179.0347, 301.0720	*Cp*	^29,30,34^
76	Caffeic acid ethyl ester	14.66	C_11_H_11_O_4_	207.0661	1.44	179.0347	*Cb, Sc*	
77	Quercetin	14.97	C_15_H_9_O_7_	301.0356	2.33	273.0407, 257.8189, 179.0346, 151.0392, 121.0288	*Sc*	^67,29,30^
78	Caffeic acid dimethyl derivative	15.01	C_11_H_11_O_4_	207.0661	1.45	16,931.0239, 151.940396, 147.069552	*Cs*	
79	Salvianolic acid F isomer	15.46	C_17_H_13_O_6_	313.0721	2.88	269.082196, 15,979.0656	*Sc, Cs*	^41^
80	Dimethylquercetin	15.49	C_17_H_13_O_7_	329.0673	3.34	314.0756, 301.0716, 179.0347, 151.0396, 121.0288	*Cb*	^29,30^
81	Trihydroxy-dimethoxyflavone	15.53	C_17_H_13_O_7_	329.0672	3.04	151.0398, 201.8020, 257.8197, 283.0612, 299.0201, 313.0722, 314.0754	*Mm*	^29,30^
82	Trihydroxylinoleic acid	16.03	C_18_H_31_O_5_	327.2183	2.75	269.0457	*Cb, Mm, Ss, Sc, Hm*	
83	Ethyl caffeate	16.14	C_11_H_11_O_4_	207.0660	0.97	179.0346	*Ss*	
84	Apigenin	16.15	C_15_H_9_O_5_	269.0459	3.35	151.0396, 117.0187	*Ss, Sc, Cr*	^40,67,29,30,32^
85	Naringenin	16.39	C_15_H_11_O_5_	271.0616	3.32	151.0397, 177.0190	*Ss, Cp*	^68,29,30^
86	Salvianolic acid F isomer	16.87	C_17_H_13_O_6_	313.0719	2.23	269.0822, 159.0658	*Sc*	^41^
87	Ethyl rosmarinate	17.23	C_20_H_19_O_8_	387.1088	2.07	179.0344, 206.9724, 359.0777	*Cb*	^20,38,39^
88	Dimethylquercetin	17.59	C_17_H_13_O_7_	329.0673	3.34	121.0291, 151.0397, 179.0350, 301.0715, 314.0756	*Ss, Cp*	^66,29,30^
89	Hesperetin	17.66	C_16_H_13_O_6_	301.0722	3.32	151.0032, 179.0343, 286.0495	*Cp*	^29,30,34^
90	Salvianolic acid F isomer	17.87	C_17_H_13_O_6_	313.0721	2.87	159.0448, 269.0821	*Cs*	^41^
91	15,16-epoxi-10S-hidroxicleroda-3,7,13(16),14 tetraeno-17, 12S; 18,19 diolido	17.94	C_20_H_19_O_6_	355.1190	2.25	311.1291	*Sh*	^55^
92	Trihydroxyoleic acid	18.13	C_18_H_33_O_5_	329.2336	2.43	171.0195, 224.7632, 250.1448	*Mm, Cb Cs*	^37^
93	Hydroxyhexadecandioic acid	18.63	C_16_H_29_O_5_	301.2025	3.32		*Cs*	^37^
94	Trihydroxy-trimethoxyflavone	18.73	C_18_H_15_O_8_	359.0766	0.28	301.6655, 314.2232, 329.0299, 344.0546	*Mm, Cb, Cp*	
95	Trihydroxy-methoxyflavanone (hesperetin isomer)	19.15	C_16_H_13_O_6_	301.0721	2.87	161.0240, 139.0032	*Cp*	^28,30^
96	trihydroxymethoxyflavone	19.23	C_16_H_11_O_6_	299.0565	3.01	151.0397, 284.0327	*So, Mm, Cr, Sh, Cp, Sd*	^69,32^
97	Cirsimaritin	19.34	C_17_H_13_O_6_	313.0724	3.19	298.0488, 283.0249	*Ss, Cr*	^70,35^
98	Isolariciresinol	19.61	C_20_H_23_O_6_	359.1502	1.95	345.1346, 344.1582, 313.0714	*Sc*	^55,31^
99	Salvianolic acid F isomer	19.77	C_7_H_13_O_6_	313.0722	3.19	269.0459, 159.8597	*Cp*	^41^
100	Rosmadial	20.03	C_20_H_23_O_5_	343.1552	1.75	299.1652, 315.1598	*Sc*	
101	Eupatorin	20.06	C_18_H_15_O_7_	343.0829	3.21	328.0595, 313.0359, 298.0125	*Cb, Mm, Ss, Cr, Cp*	^48,50^
102	Teucrol	20.3	C_17_H_15_O_6_	315.0880	3.5	179.0349, 135.0447, 161.0244	*Ss*	^51^
103	Dihydroxy-methoxyflavone	20.32	C_16_H_11_O_5_	283.0617	3.53	268.0386, 151.0034, 107.0327	*Mm, Cp*	^29,30^
104	Dihydroxy-dimethoxyflavanone	20.36	C_16_H_13_O_5_	285.0773	3.51	153.0190, 161.0453, 179.0349, 151.0397, 243.0668, 270.0535, 164.0012	*Mm*	^29,30^
105	Genkwanin	20.47	C_16_H_11_O_5_	283.0616	3.18	268.0386, 239.0922, 165.0192	*Ss, Cr, Sh*	^43^
106	Sakuranetin	20.57	C_16_H_13_O_5_	285.0771	2.81	241.1076, 165.0188, 121.0289	*Cr*	^29^
107	Octadecendioic acid	20.68	C_18_H_31_O_4_	311.2232	2.89	310.2107	*So, Sh*	^23^
108	Octadihydroxyoctadecadienoic acid	21.15	C_18_H_31_O_4_	311.2229	1.93	197.8076	*Sc*	^23^
109	Carnosol	22.2	C_20_H_25_O_4_	329.1761	2.7	285.1861	*Ss, Sc*	^38,39,54^
110	5-Epi-icetexone	22.45	C_20_H_21_O_5_	341.1396	0.88	297.1500, 299.1652	*Sc*	^56^
111	9,10-Dihydroxystearic acid	23.47	C_18_H_35_O_4_	315.2547	3.47		*Ss*	^23^

*Clinopodium brevicalyx (*Cb*), *Salvia oppositiflora* (*So*), *Minthostachys mollis* (*Mm*), *Salvia sagittata* (*Ss*), *Salvia cuspidate* (*Sc*), *Clinopodium revolutum* (*Cr*), *Clinopodium sericeum* (*Cs*), *Salvia haenkei* (*Sh*), *Hedeoma mandoniana* (*Hm*), *Clinopodium pulchellum* (*Cp*).

## Discussion

This is the first time that the phytochemical profile has been obtained for the ten Peruvian *Mentheae* (*Lamiaceae*) here reported. The botanical genera studied were *Salvia* (*Salviinae*), *Clinopodium*, *Hedeoma* and *Minthostachys* (*Menthinae*). While *Salvia* and *Clinopodium* are genera of worldwide distribution, *Hedeoma* and *Minthostachys* are American and South American genera, respectively. All *Salvia* species in this work belong to the *Salvia* subgenus *Calosphace* Benth. (Epling)^63^. Assignments were based on the search for diagnostic ions, characteristic product ions and neutral losses^19,20,25,40,41^. The fragmentation patterns shown in said references are particularly useful for this work since they are specifically directed to *Lamiaceae/Mentheae*. The phytochemical profiles of those *Mentheae* here surveyed are quite similar to their European and Asian relatives. All the species analyzed show the presence of rosmarinic acid, while, quinic acid, 3,4-dihydroxyphenyl-lactic acid “danshensu”, protocatechuic aldehyde and caffeic acid are present in most of the samples. Monocaffeoylquinic acids, also called chlorogenic acids, are also frequent but better expressed in *Minthostachys*. Dicaffeoylquinic acid was detected only in *Clinopodium revolutum*. All samples contained flavonoids with more diversity in *Minthostachys* and *Clinopodium*. Flavonoid-free aglycones predominate in several plants: In *Salvia sagitatta*, cirsimaritin is abundant^64^, while eupatorin predominates in *Clinopodium revolutum*^50^, genkwanin in *Salvia haenkei*^36^ and hesperetin in *Clinopodium pulchellum*^27^. In several plants, rosmarinic acid is the main peak: *Clinopodium brevicalyx*, *Salvia oppositiflora*, *Clinopodium sericeum* and *Hedeoma mandoniana*. Some type of salvianolic acid is present in all the samples, although in some cases, they are very small modifications of the rosmarinic acid molecule. Dimers and trimers of rosmarinic acid are present in *Clinopodium brevicalyx*, *Salvia oppositiflora*, *Salvia cuspidata*, *Clinopodium sericeum*, *Hedeoma mandoniana* and *Clinopodium pulchellum*. In *Clinopodium sericeum*, not only is the diversity of salvianolic acids important but also their abundance in salvianolic acid A, which would allow the preparation of the said substance from it^71^.

## Conclusion

Peruvian *Mentheae* are a rich source of flavonoids, phenolic acids and terpenoids. The present study involved LC-HRMS analysis of ten species. A total of 111 compounds were detected. Most of these were identified by key ion filtering strategy and comparison with literature data. This methodology can be used to the authentication and differentiation of larger numbers of *Mentheae* species: The San Marcos Herbarium, Lima-Perú, in 2017 had 108 *Mentheae*.

## Methods

### Plant material

The plants used in this study are as follows: *Clinopodium brevicalyx* Epling (Harley & Granda) (*Menthinae*) (HUT 59506), *Salvia oppositiflora* (R. and P.) (*Salviinae*) (HUT 59502), *Minthostachys mollis* Griseb. (*Menthinae*) (HUT 59766), *Salvia sagittata* R. and P. (*Salviinae*) (HUT59499), *Salvia cuspidata* subsp. *cuspidata* (R. and P.) (*Salviinae*) (HUT 59505), *Clinopodium revolutum* (R. and P.) (*Menthinae*) (HUT 58329), *Clinopodium sericeum* (Briq. et Benth) Govaerts (*Menthinae*) (HUT 58,332), *Salvia haenkei* Benth. (*Salviinae*) (HUT 59500), *Hedeoma mandoniana* Wedd. (*Menthinae*) (HUT 59763), *Clinopodium pulchellum* Kunth (Govaerts) (*Salviinae*) (HUT 59765). All of them were collected in Peru (2014–2018) by the author (C.S.) according to the procedures of the Universidad San Antonio Abad and following the guidelines of the Herbarium Truxillense of the Universidad Nacional de Trujillo (HUT)—Perú https://facbio.unitru.edu.pe. Specimens were identified and deposited by the botanist Eric Frank Rodríguez (Herbarium Truxillense).

### Sample preparation for metabolite profiling

Fifty milligrams of pulverized aerial parts were subjected to an ultrasonic bath for five minutes with 1 mL of ethanol for three times. The filtrates were evaporated in vacuo and stored at 4 °C until use.

### LC-HRMS

Chromatographic separation was performed on a Thermo Scientific Dionex Ultimate 3000 UHPLC system with an Acclaim RP C_18_ 150 × 4.6 mm × 1.8 µm chromatographic column at 25 °C and a gradient of (a) 0.1% H_2_CO_2_ in water and (b) acetonitrile: [time, % (b)]: (0.5); (5,5); (10.30); (15.30); (20,70); (25.70); (35.5) and 12 min of equilibration before each injection. The flow rate was 1 mL min^−1^, and the injection volume was 10 μL. The extracts were dissolved in 1.5 mL of methanol and filtered through 0.22 µm PTFE. For high resolution mass spectrometry, a Q-Exactive MS (Thermo Fisher Germany) equipped with electrospray ionization (ESI) in negative mode was used. The MS collection parameters were as follows: spray voltage 2500 V; capillary temperature, 400 °C. Sheath gas flowed at a rate of 75 units. Auxiliary gas flowed at 20 units. Scanning range of 100–1500 *m/z*. Resolution of 35,000. The mass tolerance threshold was 5 ppm. Data acquisition and processing were performed with XCalibur 2.3 (Thermo Fisher Scientific).

### Diagnostic ions for classification

Quinic acids derivatives: 337.0929 *p*-coumaroylquinic acid, 367.1035 feruloylquinic acid, 353.0878 caffeoylquinic acid, 515.1195 dicaffeoylquinic acid.

Phenylpropionic acids: 163.0401 *p*-coumaric acid, 179.0350 caffeic acid, 359.07772 rosmarinic acid.

Flavonoids: 253.0506 chrysin, 269.0455 apigenin, 285.0404 luteonin and kaemferol, 301.0354 quercetin.

## Supplementary Information

Supplementary Information.

## Data Availability

The datasets used and/or analyses during the current study are available from the corresponding author on reasonable request.
