# Supplementary material for: Phytochemical analysis for ten Peruvian Mentheae (Lamiaceae) by liquid chromatography associated with high resolution mass spectrometry
Source: Sci Rep. 2023 Jul 3;13:10714. doi: 10.1038/s41598-023-37830-6 (PMC10318056; doi:10.1038/s41598-023-37830-6)
Supplement: Supplementary file 1 — Supplementary Information. [file 41598_2023_37830_MOESM1_ESM.docx]

**Supplementary Material**

**Phytochemical analysis for ten Peruvian *Mentheae* (*Lamiaceae*) by liquid chromatography associated with high resolution mass spectrometry.**

**Carlos A. Serrano^a*^, Gretty K. Villena^b^, Eric F. Rodriguez^c^, Belea Calsino^d^, Michael A. Ludeña^a^, Gari V. Ccana^e^**

**^a^Laboratorio de Química Orgánica, Universidad Nacional de San Antonio Abad del Cusco, Perú. carlos.serrano@unsaac.edu.pe.**

**^b^Laboratorio de Micología y Biotecnología, Universidad Nacional Agraria La Molina, Lima-Perú.**

**^c^ Herbarium Truxillense (HUT), Universidad Nacional de Trujillo-Perú.**

**^d^Centro de Salud MINSA de Pisaq-Cusco-Perú**

**^e^Farmacia Magistral Solidaridad- Cusco-Perú.**

**Abstract**

The profile of secondary metabolites in ten members of tribe *Mentheae (Nepetoideae, Lamiaceae)* from Peru by liquid chromatography associated with high resolution mass spectrometry, is presented. Salvianolic acids and their precursors were found, particularly rosmarinic acid, caffeic acid ester derivatives, as well as a diversity of free and glycosylated flavonoids as main substances. At all, 111 structures were tentatively identified.

**Keywords:** Mentheae, Chromatography, salvianolic acids.

**Content:**

**S1 UHPLC-OT- MS Chromatograms**

**S1 UHPLC-OT- MS Chromatograms**

**
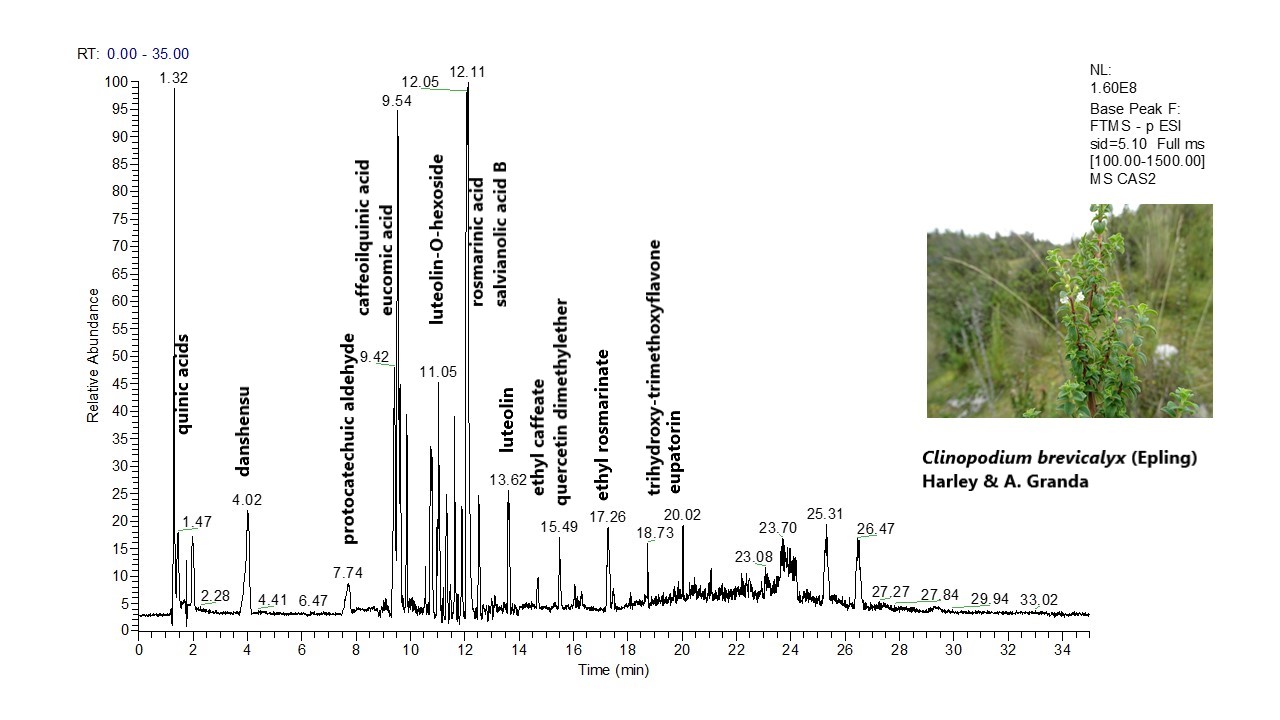

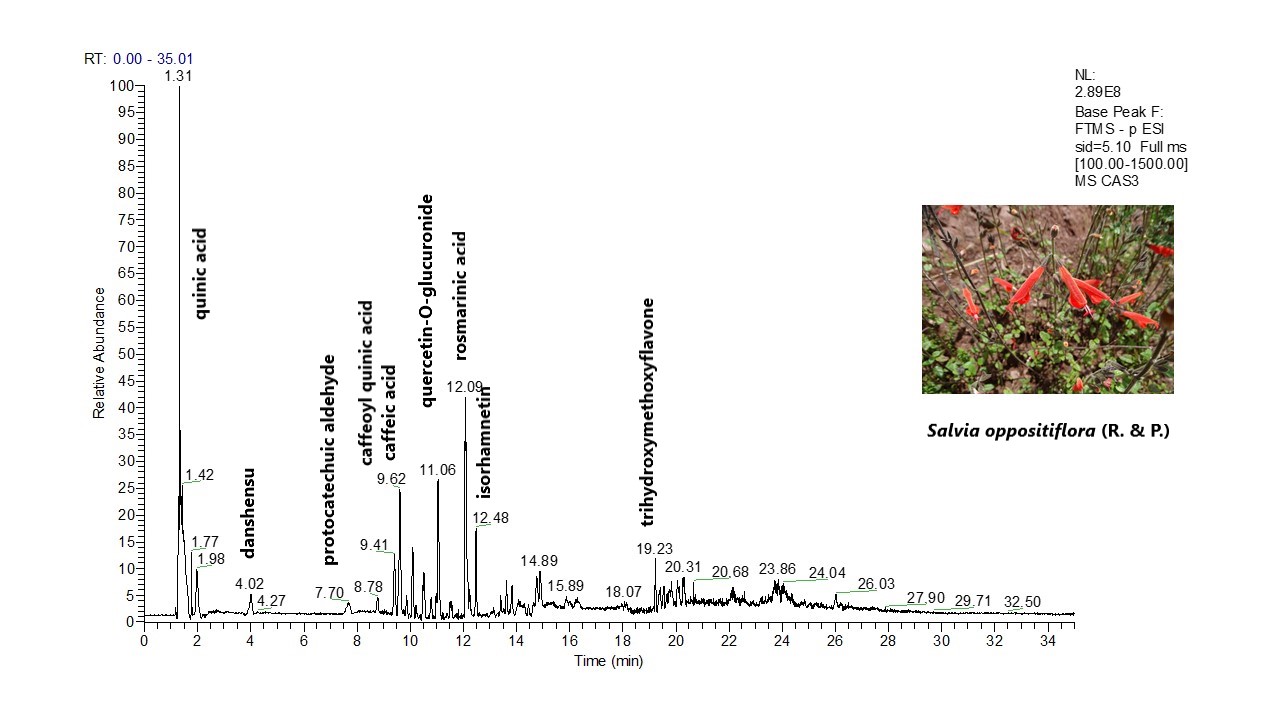
**

**
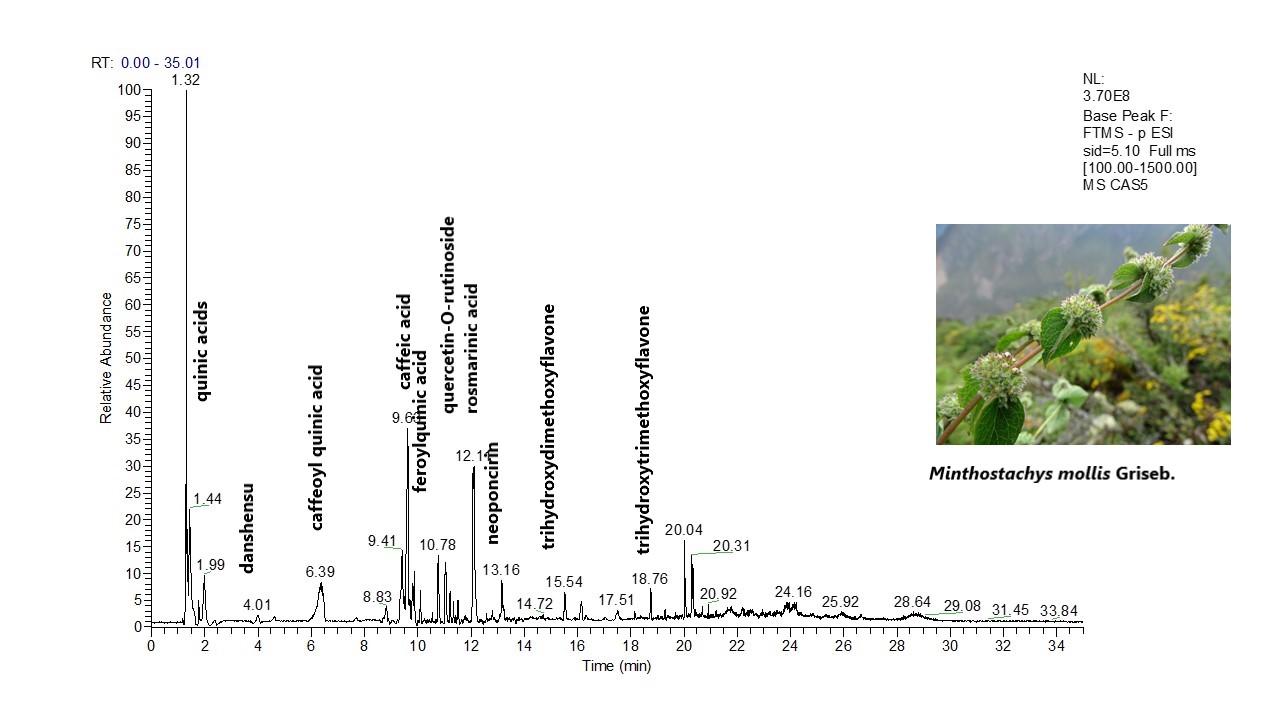
**

**
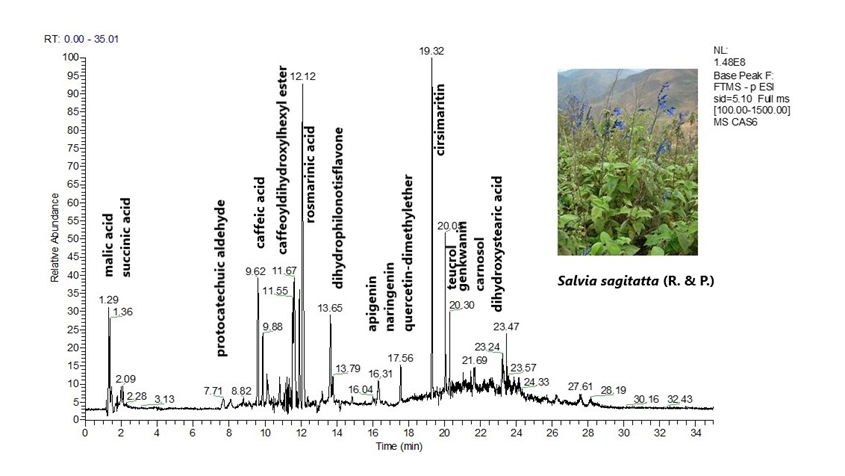
**

**
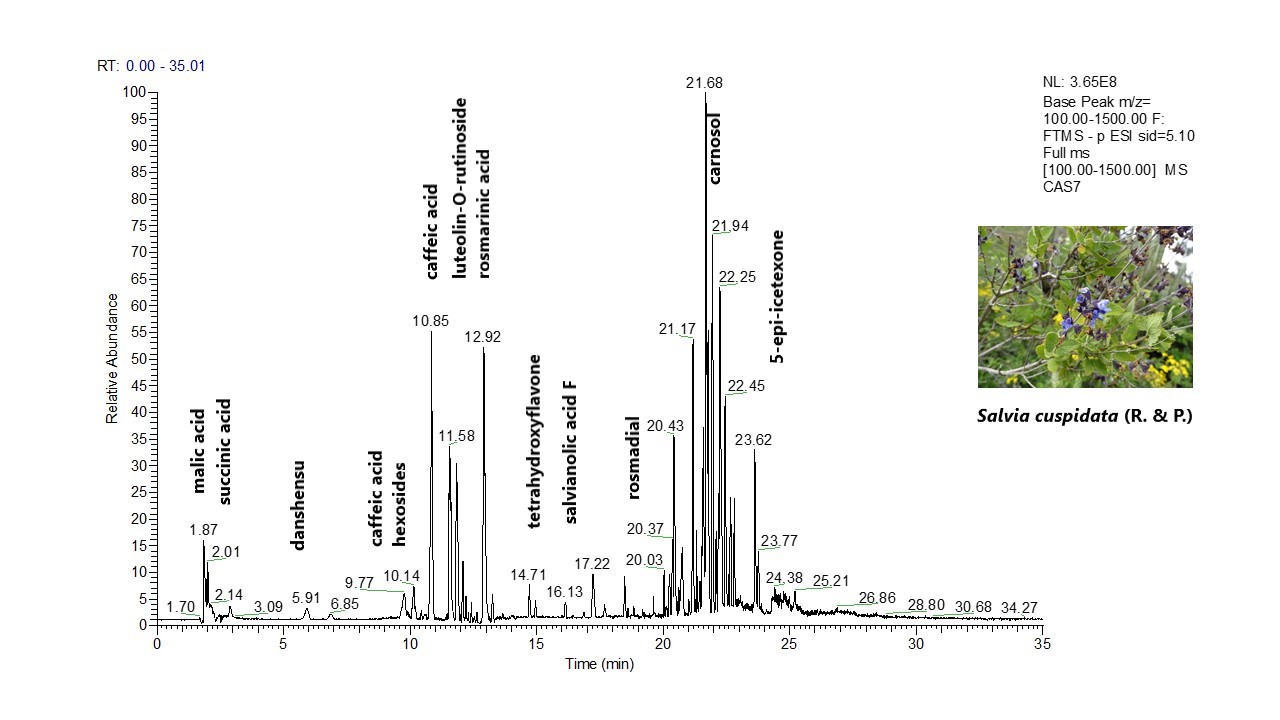

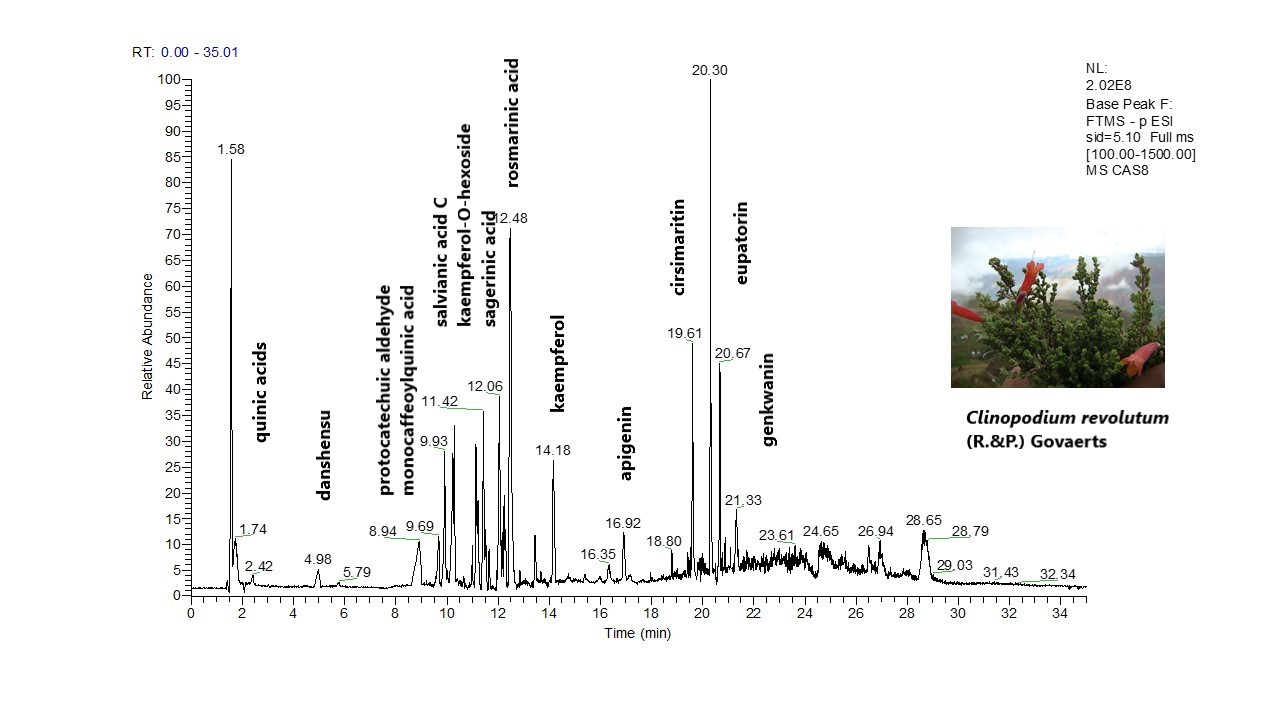

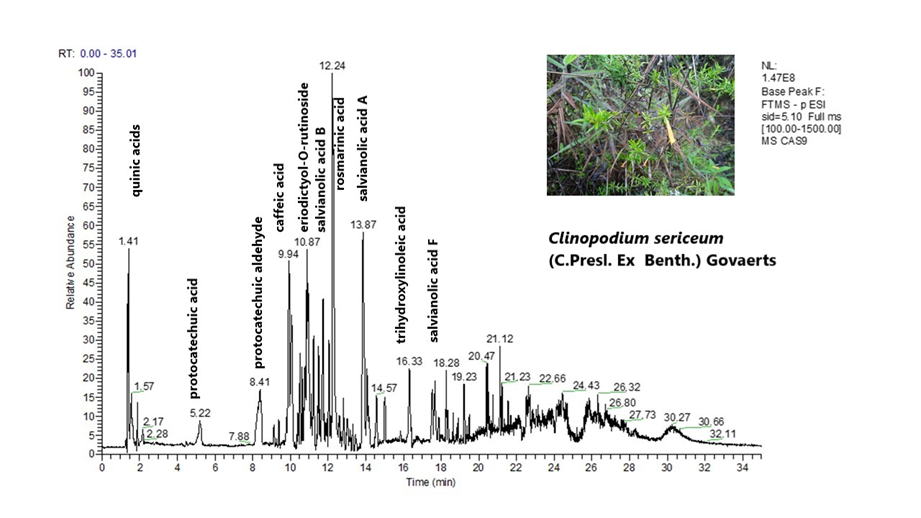
**

**
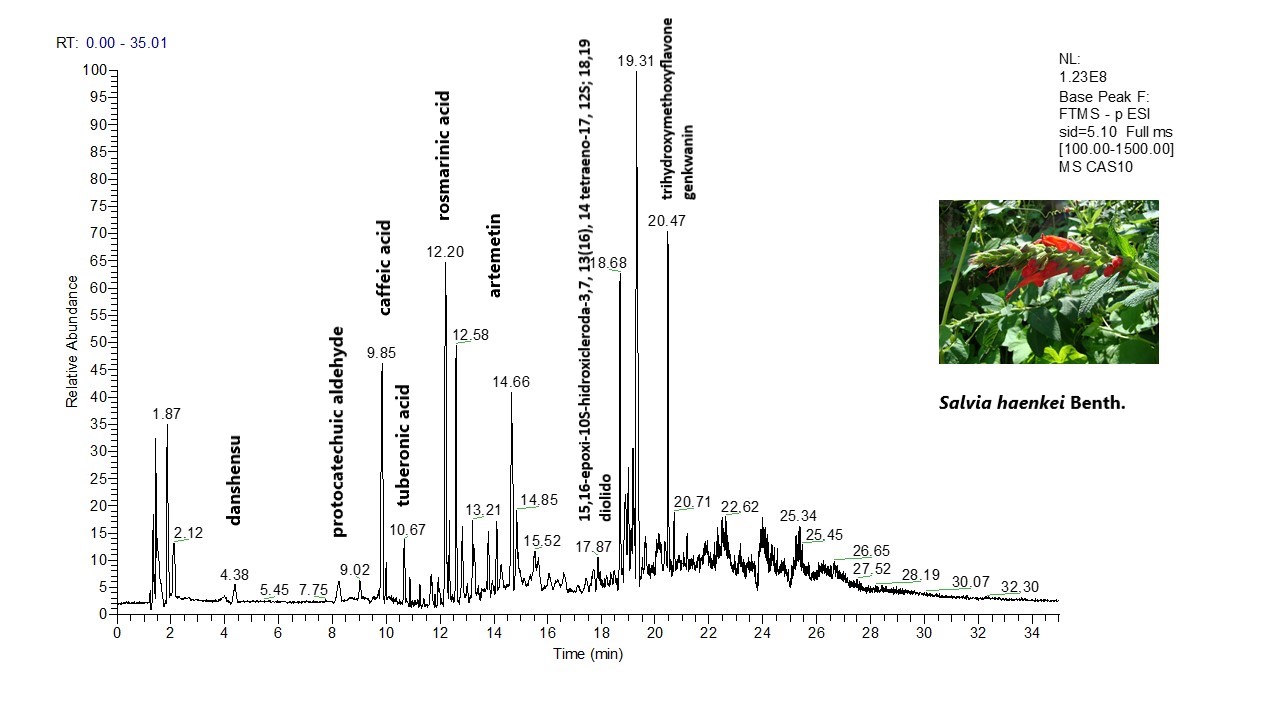
**

**
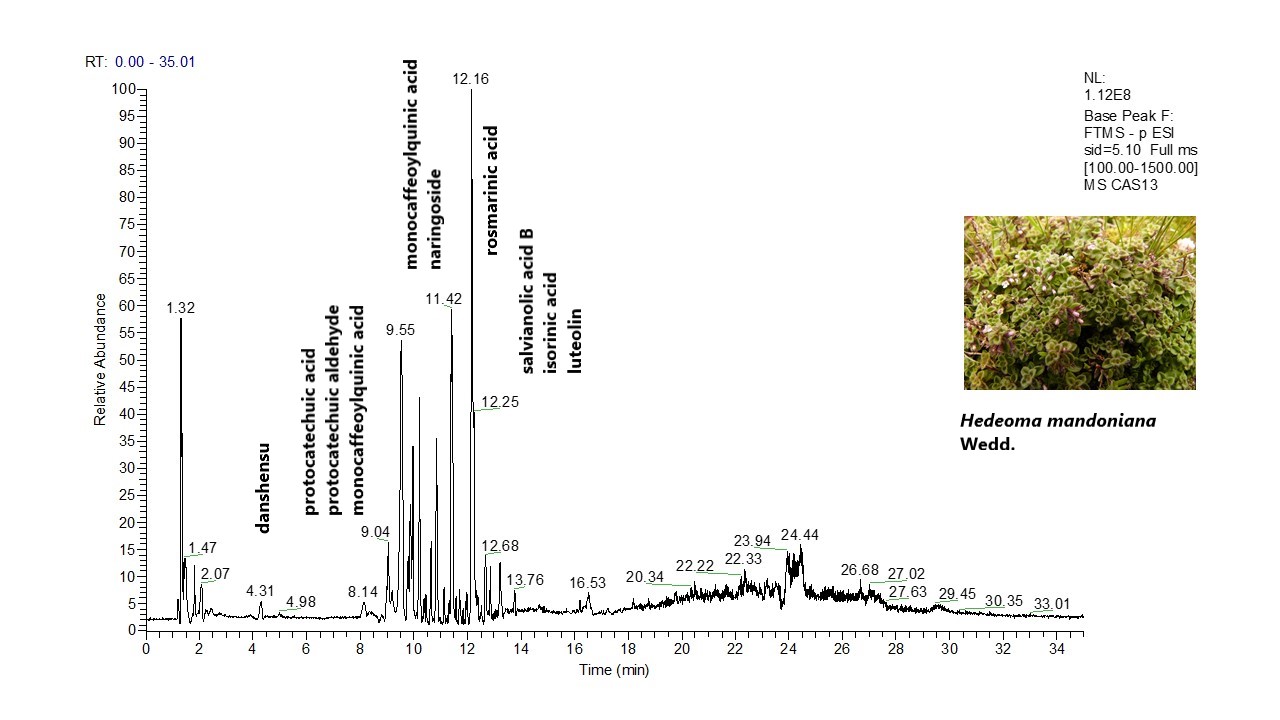
**

**
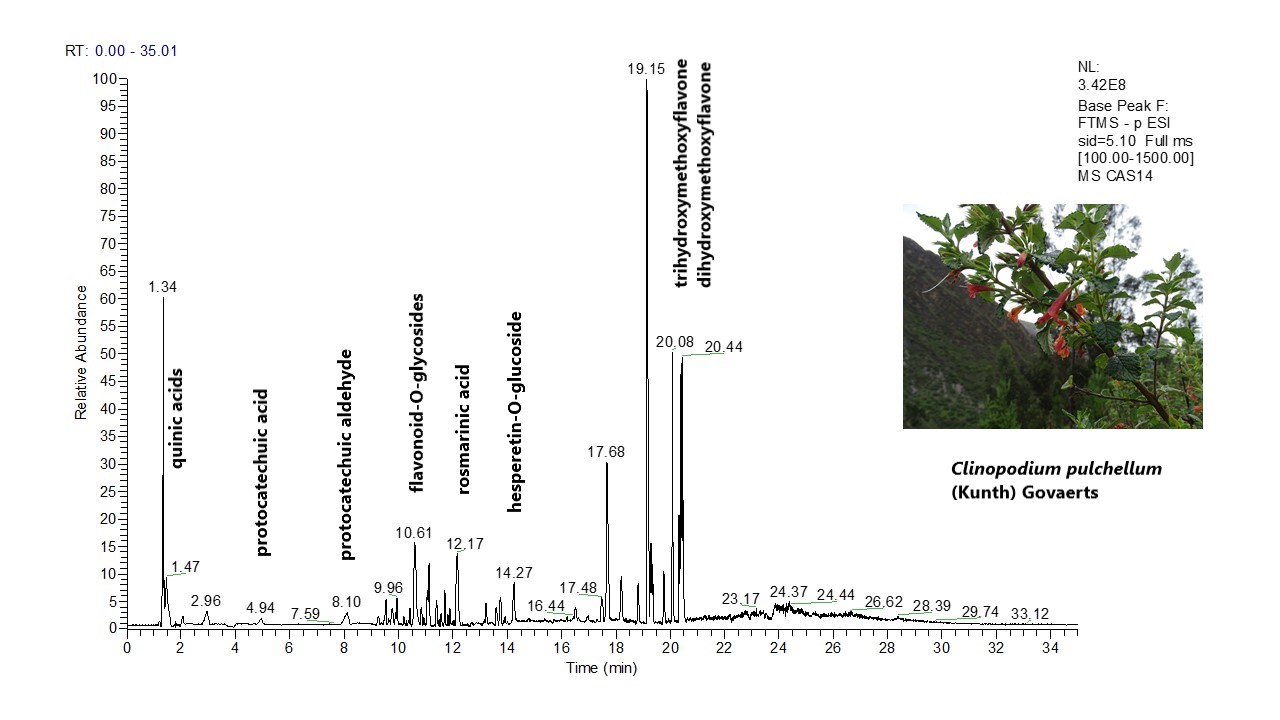
**
